# Editorial Correspondence

**Published:** 1878-03

**Authors:** P. A. McIntosh

**Affiliations:** Luraville, Fla.


### EDITORIAL CORRESPONDENCE.

LURAVILLE, FLA., February 28, 1878.

*Editor Atlanta Medical and Surgical Journal:*

Seeing so much written about belladonna, induces me to contribute my mite.

August 23d, 1877, was called at 4 o'clock, P.M., to see W. J., male, æt. 8 years. Upon examination and inquiry as to previous history, I diagnosed remittent fever. Up to this time he had had fever two days, which was not "very high," as the mother expressed it, with the usual abatements. He had taken nothing except calomel, which acted "tolerably well." All the secretions were in apparent good action; I therefore ordered nothing but that great *Dei donum*—quinia sulphas—and directed it to be taken in large doses. I left, not anticipating much trouble.

24th, 2 o'clock, A.M., was called hurriedly. Found patient in a state of intense febrile excitement, with slight delirium. Administered a dose of morphia, and used diaphoretics and arterial sedatives at intervals as I saw proper, keeping up, at the same time, the use of the quinine. The pulse was reduced from 150 to 130 during the following day. Called again at 7, P.M.; bowels somewhat distended, with tenderness on pressure. Finding there must be some intestinal obstructions, I gave a large dose sulph. magnesia, per orem, together with cathartic enemas, which produced several small, watery stools during the night.

25th. Found my patient no better. Though the fever was not so high as it had been, the bowels were more largely swollen, and now became the subject of serious consideration. Efforts to rid them of their irritating contents were persevered in during the day, with only partial success. The quinine had been continued at intervals of two hours, though there was not the slightest indication of its being absorbed. At 6 P.M., I gave one-ninth gr. ext. belladonna, by mouth, and in about two-and-a-half hours a profuse, hard, fecal discharge followed, in which was discovered the hulls of grapes, wholly undigested. Intense tinnitus aurium, produced by the quinine, was complained of. The fever gradually abated, and the patient made a speedy recovery.

I have hastily recited the above case from memory, without comments, simply to direct the attention of the profession to the twofold action of the belladonna: first, in removing the intestinal obstruction by paralyzing the nerves supplying the circular muscular fibres of the intestines; and, secondly, its peculiar action upon the nervous system, exciting a condition by which the quinine exerted its usual effect. P. A. McIntosh, M.D.

